# Editorial: Advances in chemotherapy-resistant hepatocellular carcinoma, volume II

**DOI:** 10.3389/fmed.2025.1594854

**Published:** 2025-03-27

**Authors:** Shui Liu, Yufan Liu, Mo Sha, Yang Li, Jiyao Sheng

**Affiliations:** ^1^Department of Hepatobiliary and Pancreatic Surgery, The Second Hospital of Jilin University, Jilin University, Changchun, China; ^2^Jilin Engineering Laboratory for Translational Medicine of Hepatobiliary and Pancreatic Diseases, Changchun, China; ^3^Department of Physiology, College of Basic Medical Sciences, Jilin University, Changchun, China; ^4^Yibin Institute of Jilin University, Jilin University, Yibin, China

**Keywords:** hepatocellular carcinoma, chemotherapy, chemoresistance, individualized therapy, chemoresistance mechanisms, cholangiocarcinoma, combined chemotherapy

Primary liver cancer, including hepatocellular carcinoma and cholangiocarcinoma, has a poor prognosis. According to the American Cancer Society statistics, the five-year survival rate of liver cancer is less than 20%. Chemotherapy is the preferred method to kill residual cancer cells after surgery and prolong the survival time of inoperable patients, but most cases are insensitive to chemotherapeutic agents, which leads a poor efficacy and restricts the widespread clinical application of chemotherapy in liver cancers. The reason is that liver cancer cells have primary resistance to chemotherapy drugs or acquired resistance in the process of treatment.

In recent years, the mechanism of chemotherapy resistance to liver cancer has gradually become clear with more and more studies on it, and new combined chemotherapy schemes have also been emerging. These have brought new hope for reversing the chemotherapy resistance of liver cancer. Therefore, this topic will focus on the related content of reversing the chemotherapy of primary liver cancer chemotherapy and face the clinical research and basic research of primary liver cancer chemotherapy.

The Research Topic consists of four original research papers, two reviews and one case report from prominent researchers in the field and provides readers of the journal with advanced findings in combined therapy of liver cancer, mechanisms of drug resistance and corresponding possible strategies.

Hepatocellular carcinoma (HCC) is the sixth most common cancer worldwide and the third leading cause of cancer-related deaths (1). Transarterial radioembolization (TARE), as an emerging treatment modality, has attracted increasing attention due to its significant efficacy and relatively few side effects (2). TARE treats HCC by injecting radioactive microspheres into the hepatic artery. Compared with traditional transarterial chemoembolization (TACE), TARE does not affect the blood flow of the hepatic artery or cause ischemia (3). A bibliometric analysis of TARE for HCC treatment by Hu et al. was conducted, covering 30 years of research. The study analyzed relevant literature in the Web of Science (WoS) database to reveal the research trends, hotspots, and future directions of TARE for HCC. The researchers obtained 1,110 articles related to TARE for HCC from 1993 to 2023 from the WoS database. The results showed that in terms of annual publication trends, the number of articles published each year was less than 10 from 1993 to 2006, gradually increased from 2007 to 2018, and stabilized after 2018. In terms of national publication volume and collaboration, the United States ranked first in the number of published articles, total citations, and average citation frequency. Germany, France, Italy, and China followed. In terms of international collaboration, the United States, Germany, and Italy performed prominently. In terms of institutional output and collaboration, Northwestern University published the most articles, with a total of 346. In terms of high-contributing journals, among 262 journals, those from the United States and Germany ranked first and second in terms of publication volume. In terms of characteristics of highly cited papers, the 10 most cited articles were cited 4,647 times in total, accounting for 12.28% of the total citations. These articles mainly focused on the use of ^90^Y microspheres. In terms of keyword analysis, keywords were divided into three clusters: ^90^Y microspheres for TARE, Basic research on TARE, and Clinical trial of TARE for HCC. Research hotspots gradually shifted from the use of ^90^Y microspheres to basic research and clinical trials. This study provides researchers with a comprehensive view of the current research status of TARE for HCC through bibliometric analysis methods and reveals the research trends and frontier issues in this field. This is of great value for researchers to identify key challenges, select potential partners, and determine future research directions.

Intrahepatic cholangiocarcinoma (ICC) is the second most common primary malignant tumor of the liver after HCC, and surgical resection is the only potentially curative treatment (4). However, ICC is characterized by high malignancy and a pronounced tendency for postoperative recurrence, and there is currently a lack of standardized methods for postoperative adjuvant therapy (5). A secondary study by Sun et al. reviews the postoperative adjuvant therapy strategies for ICC through a retrospective review of recent retrospective studies and clinical trials, focusing on the effectiveness, challenges, and potential development directions of the treatment. The article points out that for the beneficiaries of postoperative adjuvant therapy, patients with lymph node metastasis and positive surgical margins are currently considered ideal candidates for adjuvant therapy. Other high-risk factors include multiple tumors, low differentiation, tumor size over 5 cm, vascular and nerve invasion, and elevated CA19-9 levels. Regarding chemotherapy, although most retrospective studies have shown that adjuvant chemotherapy is beneficial for high-risk ICC patients, there is currently no consensus. The interim results of the BILCAP trial indicate that capecitabine has become the recommended drug for postoperative adjuvant therapy of ICC and CCA (6). However, the selection of chemotherapy regimens still requires further prospective studies. Regarding postoperative adjuvant radiotherapy for ICC, there is currently a lack of data supporting postoperative adjuvant radiotherapy for ICC. With the advancement of medical science and the in-depth understanding of the molecular level of ICC, more effective treatment methods may be provided for ICC in the future.

**Figure 1:**
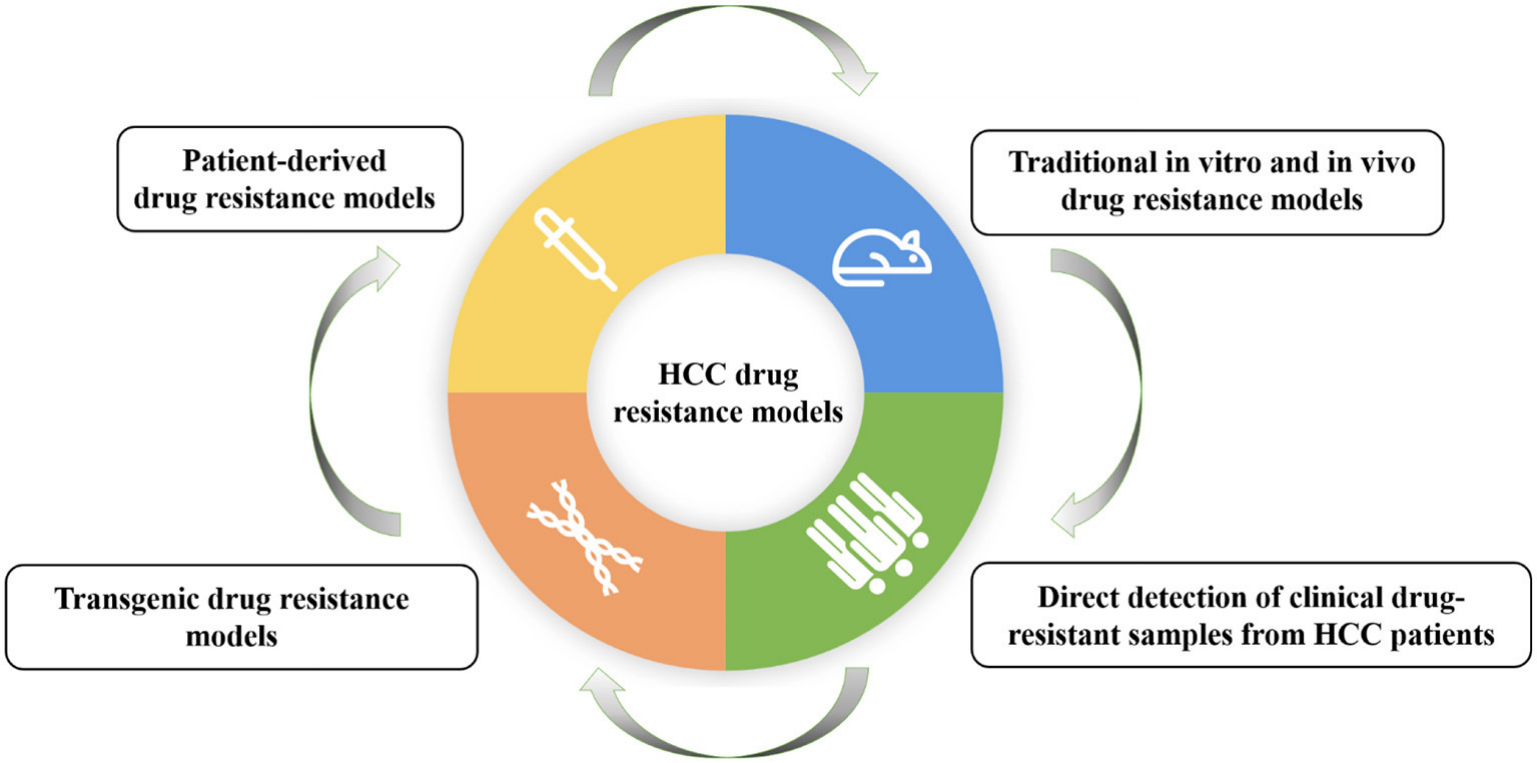
Classification of hepatocellular carcinoma drug resistance models.

Another study by Xiang et al. mainly focuses on the research progress of drug resistance models for HCC. The researchers comprehensively review the establishment methods and applications of HCC drug resistance models, including traditional in vitro and in vivo drug resistance models, patient-derived drug resistance models, and direct detection of clinical drug-resistant samples from HCC patients, and transgenic drug resistance models (Figure 1). It also discusses the main drug resistance mechanisms discovered based on these models and provides a model basis for possible future personalized treatment. They found that traditional drug resistance models are easier to establish and the corresponding experimental results have higher stability and reproducibility. However, individual differences in HCC gene expression could be reflected; patient-derived models retain more individual characteristics and are crucial for studying multiple drug resistance pathways related to different clinical subtypes; direct detection of clinical drug resistance samples may be a simpler method for screening drug resistance genes; gene editing methods can be used to generate gene-engineered cell lines or animal models with resistance to specific drugs. Therefore, the appropriate drug resistance model should be selected based on the research purpose and actual research environment.

He et al. focus on the radiological and clinical factors that influence untreatable progression (UP) and time to UP (TTUP) in HCC patients after local regional interventional therapy. This study included HCC patients who received drug-eluting bead TACE (DEB-TACE) treatment in the hospital from January 2017 to December 2022. Multivariate logistic regression analysis shows that age (OR: 0.950, *p* = 0.044), initial response (OR: 0.177, *p* = 0.020), and treatment regimen (OR: 7.133, *p* = 0.007) are three independent risk factors of UP, and the treatment regimen had the greatest impact on the occurrence of UP. Multivariate Cox regression analysis shows that total bilirubin (HR: 1.029, *p* = 0.002), tumor distribution (HR: 1.752, *p* = 0.034), SACE classification (HR: 0.668, *p* = 0.043), tumor number (HR: 1.130, *p* = 0.004), initial response (HR: 0.539, *p* = 0.019), and treatment regimen (HR: 4.615, *p* < 0.001) are independent variables affecting TTUP. The treatment regimen had the greatest impact on TTUP. The TTUP of patients with initial response was significantly longer than that of patients without initial response (329 days vs. 166 days, *p* < 0.05). The TTUP of patients with the best response was also significantly longer than that of patients without the best response (279 days vs. 130 days, *p* < 0.05). Subjective Angiographic Chemoembolization Endpoint (SACE) classification significantly affected TTUP, with TTUP of patients in SACE III and IV grades significantly longer than those in grades I and II (269 days and 533 days vs. 70 days and 144 days, *p* < 0.05). Tumor distribution significantly affected TTUP, with TTUP of multi-lobed tumors shorter than that of single-lobed tumors.

In recent years, targeted therapy and immunotherapy have made significant progress in the treatment of HCC and ICC (7, 8). For instance, drugs targeting FGFR2, IDH1/2, etc. have shown good efficacy in clinical trials (9–11). Immunotherapy, especially immune checkpoint inhibitors, such as PD-1/PD-L1 inhibitors, has demonstrated great potential in the treatment of HCC and ICC (12, 13). Local regional interventional therapy (such as TACE, ablation, and radioembolization) also plays an important role in the treatment of advanced liver cancer. By optimizing treatment plans, such as TACE combined with ablation therapy, the treatment effect can be improved and the survival period of patients can be prolonged. In addition, image-guided precise interventional therapy can reduce complications and improve patient tolerance. Based on the patient's genetic characteristics, tumor biological behavior and clinical features, personalized treatment plans will become the future development direction. Through the collaboration of multidisciplinary teams, considering the individual differences of patients comprehensively, the most suitable treatment strategy can be selected to improve treatment effect and the quality of life of patients. With the continuous advancement of technology and the deepening of multidisciplinary collaboration, the diagnosis and treatment of liver cancer will witness more breakthroughs and progress in the future.
